# A novel immunohistochemical score predicts the postoperative prognosis of gastric cancer patients

**DOI:** 10.1186/s12957-023-03113-7

**Published:** 2023-07-26

**Authors:** Feng Liu, Xiaoyang Wu, Weiping Wang, Jun Chang

**Affiliations:** 1 Department of Gastrointestinal Surgery, Affiliated Kunshan Hospital of Jiangsu University, Suzhou, Jiangsu 215300 People’s Republic of China; 2 Department of General Surgery, Kunshan Second People’s Hospital, Suzhou, 215300 People’s Republic of China

**Keywords:** Gastric cancer, Immunohistochemical, Prognosis, Prediction system

## Abstract

**Background and aim:**

Immunohistochemistry indicators are increasingly being used to predict the survival prognosis of cancer patients after surgery. This study aimed to combine some markers to establish an immunohistochemical score (MSI-P53-Ki-67[MPK]) and stratify postoperative patients with gastric cancer according to the score.

**Methods:**

We used 245 patients who underwent surgery at one center as the training cohort and 111 patients from another center as the validation cohort. All patients were treated between January 2012 and June 2018. The training cohort was screened for prognostic factors, and MPK scores were established using univariate and multifactorial COX risk proportional models. Patients were prognostically stratified according to the MPK score after gastrectomy for gastric cancer. Overall survival (OS) and recurrence-free survival (RFS) rates were compared among low-, intermediate-, and high-risk groups using the Kaplan–Meier method, and survival curves were plotted. Finally, the MPK score was validated using the validation cohort.

**Results:**

In the training group, there were statistically significant differences in OS and RFS in the low, medium, and high-risk groups (*P* < 0.001). Thirty patients were in the high-risk group (12.2%). The median survival times of the three groups were 64.0, 44.0, and 23.0, respectively, and median times to recurrence were 54.0, 35.0, and 16.0 months, respectively. In the validation group, the prognosis in the three risk groups remained significantly different (*P* < 0.001).

**Conclusions:**

The novel MPK score could effectively predict the postoperative OS and RFS of gastric cancer patients, risk-stratify postoperative patients, and identify postoperative high-risk patients for refined management.

**Supplementary Information:**

The online version contains supplementary material available at 10.1186/s12957-023-03113-7.

## Introduction

Gastric cancer (GC) is one of the most severe digestive tract tumors globally, with many new cases added each year [1, 2]. Surgical resection is one of the best treatment methods for GC patients that can be curatively resected. Most patients can achieve a good prognosis under our comprehensive treatment model based on surgery and adjuvant chemotherapy. However, due to the heterogeneity of tumors [3], some patients are prone to recurrence or metastasis after surgery. Because the prognosis of this group of patients is not satisfactory, we hope that this group of patients can be screened out for more refined management.

Some immunohistochemical indicators play a vital role in tumor prediction, and Dudley et al. [4] suggested that microsatellite instability could be used as a biomarker for PD-1 blockers in different tumors (colorectal, endometrial, cervical, esophageal, skin, and breast cancer) and that MSI plays a more significant role in precision medicine. Yang et al. [5] also concluded that MSI was associated with tumor biology and suggested that MSI could be a key predictor of tumor malignancy, outcome, and prognosis; Zeng et al. [6] conducted a comprehensive search of relevant research and used the TCGA dataset and found that positive expression of ki-67 correlated with OS and DFS in osteosarcoma. Kirsch et al. [7] concluded that the P53 oncogene plays an essential role in the prognosis of certain tumors. Therefore, it is crucial to use these indicators to stratify postoperative patients and further treat high-risk patients.

The TNM staging system has been widely used to predict GC prediction, but the staging is for patients receiving different treatments, and even at the same stage, the prognosis of patients may be completely different [8]. Pang et al. [9] used the preoperative inflammatory, nutritional, and tumor marker index to develop a model that can predict the overall survival of GC patients and obtain good discrimination. However, their model relied mainly on preoperative indices, which are dynamic, and the prediction may be biased. Therefore, it is essential to identify new additional predictive markers, and there is an urgent need for scoring systems that can discriminate postoperative patients by which high-risk patients can be identified, to improve prognostic prediction and provide appropriate treatment.

Some current immunohistochemical metrics that play a vital prognostic role, including ki-67, MSI, CD44, and Her-2 [10], have proven to have a robust predictive role for patient prognosis in different studies. Therefore, we combined some of these factors to construct a new immunohistochemical score (MSI-P53-Ki-67) [MPK] to identify high-risk postoperative patients.

## Materials and methods

### Patient selection

We enrolled 356 GC patients who underwent gastrectomy from two centers, 256 from Affiliated Kunshan Hospital of Jiangsu University and served as the training group and 111 from Kunshan Second People’s Hospital and served as the validation group; all enrolled from January 2012 to June 2018. Strict inclusion and exclusion criteria were applied to select patients from both centers.

Inclusion criteria are as follows: (1) patients treated with radical surgery, (2) patients’ postoperative pathology reports were jointly determined to be gastric cancer by two experienced pathologists, (3) patients’ preoperative American Society of Anesthesiology (ASA) score ≤ II, (4) Eastern Tumor Collaborative Group score ≤ 2 (5) were first tumor findings, (6) no treatment prior to admission, and (7) all patients underwent immunohistochemistry—the results of which were reported by two experienced pathologists.

Exclusion criteria are as follows: (1) incomplete follow-up information, (2) initial diagnosis was multiple systemic metastases, (3) severe postoperative complications, and (4) received adjuvant chemotherapy. This retrospective study was approved by the ethics committees of Affiliated Kunshan Hospital of Jiangsu University and Kunshan Second People’s Hospital and followed the Declaration of Helsinki. All patients signed an informed consent form.

### Surgical procedures

All patients underwent a rigorous preoperative evaluation to meet the preoperative criteria for surgery. All patients underwent preoperative laboratory tests such as complete blood count, liver and kidney function, coagulation function, and preoperative imaging examinations including abdominal ultrasound, enhanced abdominal CT, and abdominal magnetic resonance imaging (MRI) for comprehensive consideration of resectability. The surgical approaches were adjusted based on the locations of the tumors.

Total gastrectomy was considered if the tumor was close to the cardia and the upper third of the stomach. Tumors growing in the body of the stomach were treated with distal gastrectomy or total gastrectomy, depending on the case; tumors growing in the distal part of the stomach (sinus) were treated with distal gastrectomy. Lymphadenectomy plays a crucial role in gastrectomy for gastric cancer, aiming to excise both the gastric tumor and the associated lymph nodes. The extent and meticulousness of lymphatic dissection are determined by several factors, including tumor location, type, stage, and the patient’s overall condition. Commonly involved lymph nodes encompass peripyloric lymph nodes (near the pylorus), fundic lymph nodes (near the fundus), and those adjacent to the greater and lesser curvatures of the stomach. The primary objective of lymphatic clearance is to ensure thorough removal of the affected lymph nodes, enabling accurate disease assessment, and minimizing the risk of tumor recurrence and metastasis. Surgeons employ specific anatomical landmarks, image guidance, or lymph node visualization techniques during the procedure to facilitate precise localization and removal of lymph nodes. An experienced surgical team performed all surgical procedures. The surgical teams consisted of ultrasonographers, gastrointestinal surgeons, oncologists, and pathologists.

### Immunohistochemistry evaluation

For the discrimination of microsatellite instability, we used single nucleotide repeats of the five markers BAT-26, BAT-25, NR-24, NR-21, and NR-27, which we defined as MSI-H when ≥ 2 markers showed instability, according to the definition (National Cancer Institute on Cancer MSI).

P53 wild-type expression is primarily involved in cell cycle arrest, apoptosis, or DNA repair by acting as a homotetrameric transcription factor, binding to specific DNA sequences, and regulating gene expression [11]. For p53, monoclonal mouse anti-human p53 protein clone DO-7 DAKO (code N1581) was used. For Ki-67, we used monoclonal mouse anti-human Ki-67 antigen clone MIB-1 DAKO (code N1633). According to the kit manual recommendations, the initial incubation time for these primary antibodies was 25 min at room temperature. After washing with TBS, the slides are incubated at room temperature with a layer of biotinylated goat anti-rabbit/mouse immunoglobulin (secondary antibody) in phosphate-buffered saline (PBS) containing stabilized protein. An additional section was stained as a negative control for each of the above markers without applying primary antibodies. Next, streptavidin conjugated with horseradish peroxidase is added to the buffer (incubation time: 25 min). After washing and adding a drop of substrate-chromogen, incubate for 10 min, i.e., cross-stain with HandE (DAKO LSAB2 system-HRP). When > 10% of the cells’ nuclei showed staining for p53, it was considered to be positive (overexpressed). For Ki-67, > 50% of positive staining in the nucleus was defined as positive staining. The cutoff values for Ki-67 and P53 staining were chosen based on the most common cutoff values used by most researchers in the previous literature [12, 13].

### Establishment of MPK scores

Using multivariate COX regression analysis, the HR of each immunohistochemical factor was obtained. Based on the obtained immunohistochemical indices, each index was assigned a value based on the comparison between HR values. A score of 3 was assigned when the MSI status was MSI-high and 0 when it was MSS/MSI-low; a score of 2 was assigned when P53 was + and 0 when P53 was -; a score of 2 was assigned when Ki-67 expression was ≥ 50% and 0 when it was < 50%. The scores obtained for each index were then added to obtain the total score, which is the MPK score. We ranked the patients according to their obtained MPK scores and divided all patients into three risk groups: low-risk group with a score of 0; moderate risk group with a score of 2–5, and high-risk group with a score of 7.

### Variables

Based on the patients’ basic characteristics as well as tumor characteristics and immunohistochemical features, we collected 14 variables, including gender, age, American Society of Anesthesiology (ASA) score, ECOG PS, T/N-stage and TNM-stage, tumor size, differentiation, vascular invasion, P53 ( ±), Ki-67, and MSI status.

### Definitions

The imaging physician determines the TNM stage of each patient based on enhanced computed tomography (CT) and abdominal magnetic resonance imaging (MRI). We followed up with each postoperative patient with a process performed by two followers, every 3 months during the first year after discharge and every 6 months starting in the next year. At each follow-up visit, enhanced CT and abdominal MRI were performed, and PET-CT was performed for necessary patients and laboratory tests such as liver function, kidney function, and tumor markers. Overall survival (OS) was defined as the first postoperative day to the date of death or the last follow-up date, and recurrence-free survival (RFS) was defined as the period between the first postoperative day to the date of recurrence or the last follow-up date for those without recurrence (*recurrence* was defined as the detection of a new lesion on postoperative imaging). The follow-up period was up to February 30, 2022. The median duration of follow-up for all patients was 52.0 months, with a 95% CI of 45.4–58.6 months.

### Data analysis

All categorical variables were tested by the chi-square test or Fisher’s exact test. Survival curves were plotted using the Kaplan–Meier method of survival curves, and statistical tests were performed using the log-rank method. Variables with *P* values < 0.05 on univariate regression analysis were included in the multivariate regression analysis and screened for prognostic capability.

Results were based on the hazard ratio of OS in the multifactorial COX risk model and rounded to the nearest integer for further analysis. We used this method to obtain the weights of each immunohistochemical index, and the scores of each immunohistochemical index were summed to obtain the MSI-P53-Ki-67 (MPK) score. All postoperative gastric cancer patients were also divided into low, moderate, and high-risk groups according to the MPK scoring system.

Data were analyzed using SPSS 25.0 (IBM, Armonk, New York, USA), and calculated *P* values < 0.05 (two-tail) were considered statistically significant. All survival curves were plotted using R software (R Project for Statistical Computing, Vienna, Austria; version 4.0.5). Before the study was conducted, sample size estimation was performed using PASS (Version: 11.0).

## Results

### Baseline information of patients in the training and validation groups

There were a total of 245 patients in the training group, of whom 204 (83.3%) were males, 108 (44.1%) were older than 60 years, and 217 (88.9%) were ASA grade I. The number of patients in each TNM stage was similar, with 107 (43.7%) having P53 ( +) and 116 (47.3%) having Ki-67 ≥ 50%. There were 50 patients with high MSI (MSI-H), accounting for 20.4%. There were 111 patients in the validation group. Among them, 42 patients were P53 ( +) accounting for 37.8%, 46 patients were Ki-67 ≥ 50%, accounting for 41.4%, and 21 patients were MSI-H accounting for 18.9%, and there was no statistical difference in the variables between the two groups of training and validation (Table 1). Additionally, we generated baseline characteristics tables based on different risk groups. In the training cohort, there were 111, 104, and 30 individuals in the low, intermediate, and high-risk groups, respectively. Significant statistical differences were observed among the groups in terms of gender, TNM stage, differentiation, P53, Ki-67, MSI status, recurrence number, and recurrence model variables (Supplementary Table 2).

**Table 1:** Baseline characteristics of patients with gastric cancer undergoing gastrectomy in the training and validation cohorts (*n* = 356)

	**Training cohort (*n* = 245)**	**Validation cohort (*n* = 111)**	***p* value**
Gender (%)			0.929
Male	204 (83.3)	92 (82.9)	
Female	41 (16.7)	19 (17.1)	
Age (%)			0.566
< 60 years	137 (55.9)	58 (52.3)	
≥ 60 years	108 (44.1)	53 (47.7)	
ASA (%)			0.307
I	217 (88.6)	94 (84.7)	
II	28 (11.4)	17 (15.3)	
ECOG PS (%)			0.888
0	193 (78.8)	89 (80.2)	
1	52 (21.2)	22 (19.8)	
Depth of tumor invasion (%)			0.721
T1	49 (20.0)	20 (18.0)	
T2	25 (10.2)	11 (9.9)	
T3	126 (51.4)	59 (53.2)	
T4	45 (18.4)	21 (18.9)	
*N* status (%)			0.132
N0	56 (22.9)	28 (25.2)	
N1	63 (25.7)	23 (20.7)	
N2	62 (25.3)	25 (22.5)	
N3	64 (26.1)	35 (31.5)	
TNM stage (%)			0.081
I stage	61 (24.9)	40 (36.0)	
II stage	38 (15.5)	34 (30.6)	
III stage	81 (33.1)	20 (18.0)	
IV stage	65 (26.5)	17 (15.3)	
Tumor size (%)			1.000
< 5 cm	110 (44.9)	50 (45.0)	
≥ 5 cm	135 (55.1)	61 (55.0)	
Differentiation (%)			0.547
High or moderate	86 (35.1)	35 (31.5)	
Poor or no	159 (64.9)	76 (68.5)	
Vascular invasion (%)			0.809
No	82 (33.5)	39 (35.1)	
Yes	163 (66.5)	72 (64.9)	
P53 (%)			0.354
No	138 (56.3)	69 (62.2)	
Yes	107 (43.7)	42 (37.8)	
Ki-67 (%)			0.304
< 50%	129 (52.7)	65 (58.6)	
≥ 50%	116 (47.3)	46 (41.4)	
MSI status (%)			0.777
MSS/MSI-low	195 (79.6)	90 (81.1)	
MSI-high	50 (20.4)	21 (18.9)	
Follow-up period			
Month (95%CI)	49.0 (41.4–55.6)	56.0 (47.6–68.8)	0.130
Recurrence model			0.513
Local recurrence	44 (18.0)	21 (18.9)	
Lymph node metastasis	33 (13.5)	17 (15.3)	
Intra-abdominal metastasis	70 (28.6)	34 (30.6)	
Other organ metastases	39 (15.9)	14 (12.6)	

TNM stages are according to AJCC 8th edition

*ASA* American Society of Anesthesiologists, *ECOG* Eastern Cooperative Oncology Group, *PS* Performance status, *MSI* Microsatellite instability, *MSS* Microsatellite stable

### Univariate and multivariate Cox regression analysis for prognostic factors of postoperative gastric cancer patients and the construction of MPK scores

We first performed univariate analysis and obtained variables with *P* < 0.05 to be included in the multifactorial analysis. TNM stage (HR = 1.584 [1.365–1.837]), P53 (HR = 1.862 [1.205–2.880], Ki-67 (HR = 1.606 [1.055–2.444]), and MSI status (HR = 2.771 [1.864–4.121]) were the four variables that were prognostic of the OS (Table 2). After risk-stratifying the patients based on the MPK scores, 111 (45.3%) patients were in the low-risk group, 104 (42.4%) patients were in the moderate-risk group, and 30 patients (12.2%) were in the high-risk group in the training cohort (Table 3). After constructing the scores, the MPK scores were included in univariate and multifactorial COX regression analyses, and the multifactorial results showed an HR = 2.899 [2.121–3.846] for the MPK scores (Supplementary Table 1).

**Table 2:** Univariate and multivariate analysis of overall survival in gastric cancer (GC) patients who underwent gastrectomy in the training cohort

	**Univariate analysis**			**Multivariate analysis**		
	*P*	HR	95% confidence interval	*P*	HR	95% confidence interval
**Gender** Male/female	0.921	1.020	0.692–1.504			
**Age** > 60 years/ ≤ 60 years	0.181	0.817	0.608–1.099			
**ASA** II/I	0.714	0.920	0.587–1.440			
**ECOG PS** 1/0	0.984	1.004	0.706–1.427			
**TNM stage**	**< 0.001**	1.711	1.490–1.965	< 0.001	1.584	1.365–1.837
**Tumor size**	**0.002**	1.594	1.182–2.149	0.119	1.283	0.938–1.755
> 5.0 cm/ ≤ 5.0 cm						
**Vascular invasion**	0.427	1.131	0.835–1.531			
Yes/No						
**P53**	**< 0.001**	2.298	1.682–3.140	0.005	1.862	1.205–2.880
±						
**Ki-67** ≥ / <	**< 0.001**	2.614	1.923–3.555	0.027	1.606	1.055–2.444
**MSI status** MSI-high/MSS or MSI-low	** < 0.001**	2.710	1.880–3.907	< 0.001	2.771	1.864–4.121

TNM stages are according to AJCC 8th edition

*ASA* American Society of Anesthesiologists, *ECOG* Eastern Cooperative Oncology Group, *PS* performance status, *MSI* microsatellite instability, *MSS* microsatellite stable, *HR* hazard ratio

**Table 3:** Components of the immunohistochemistry scores (MPK)

Risk factor	Score
**MSI status**	
MSI-high	3
MSS/MSI-low	0
**P53**	
+	2
−	0
**Ki-67**	
≥ 50%	2
< 50%	0

Low-risk group: score 0; moderate-risk group: scores 2–5; high-risk group: score 7

### The OS and RFS of the different risk groups in the training cohort

**Fig. 1:**
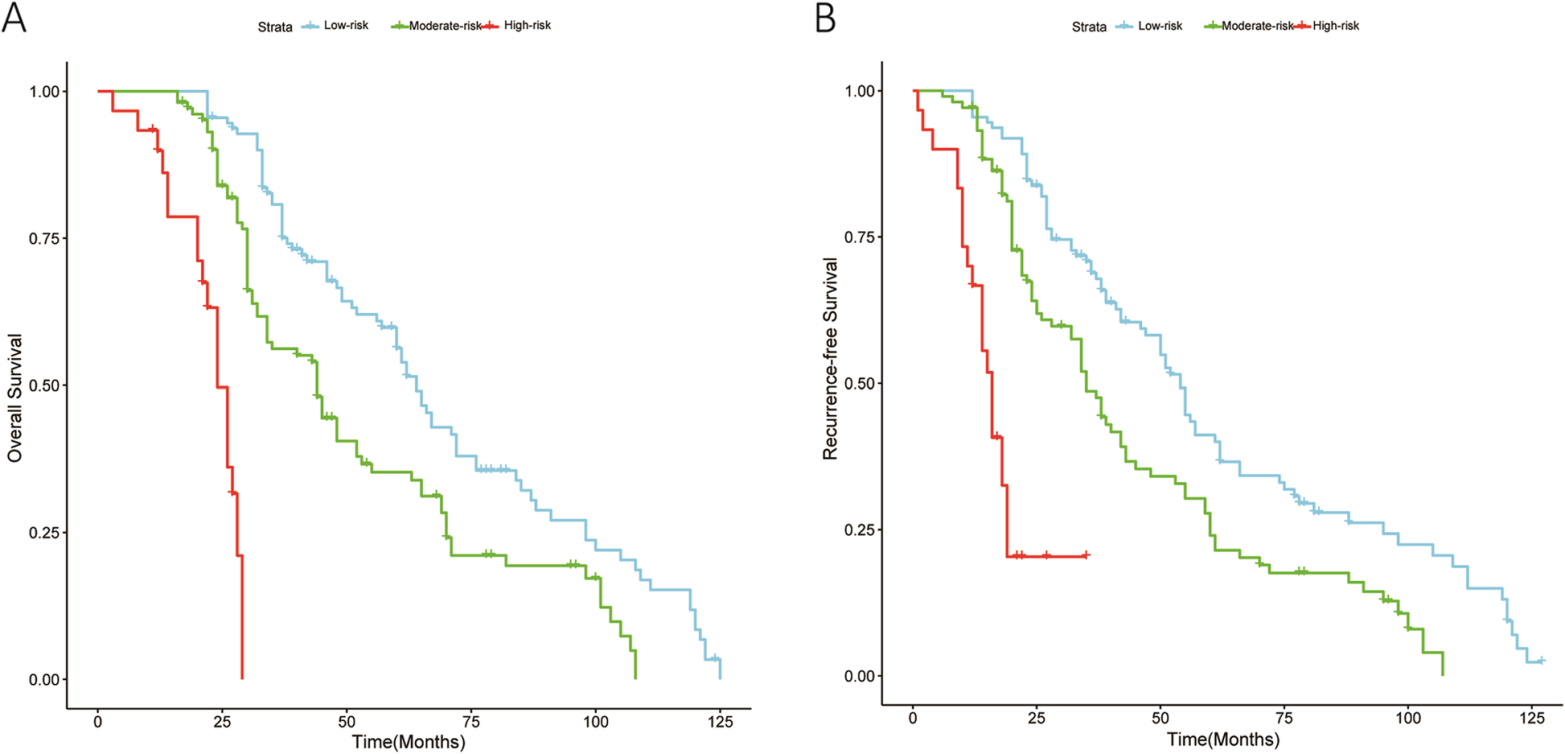
Overall survival (OS) and recurrence-free survival (RFS) of gastric cancer patients in the training cohort based on MPK scores after surgery. **A** Overall survival. **B** Recurrence-free survival. In the low-risk group, the 1-, 3-, and 5-year overall survival rates were 100.0%, 80.7%, and 56.3%, with a median survival time of 64.0 months. For recurrence-free survival, 95.5%, 68.8%, and 40.1% of patients in the low-risk group survived recurrence-free at 1, 3, and 5 years, with a median time to recurrence of 54.0 months. In the moderate-risk group, the 1-, 3-, and 5-year overall survival rates were 98.1%, 56.2%, and 33.9%, with a median survival time of 44.0 months. The corresponding recurrence-free survival rates were 93.2%, 48.7%, and 24.0%, with a median time to recurrence of 35.0 months. In the high-risk group, the 1-, 3-, and 5-year overall survival rates were 78.6%, 0.0%, and 0.0%, with a median survival time of 23 months. The recurrence-free survival rates in this group were 66.7%, 0.0%, and 0.0%, with a median time to recurrence of 16.0 months. Statistically significant differences were observed in survival and recurrence rates among the three groups (*P* < 0.001)

According to the MPK score, patients were divided into low, moderate, and high-risk groups. In Fig. 1A, there was a statistically significant difference in prognosis between the three groups (*P* < 0.001). The 1-, 3- and 5-year overall survival rates were 100.0%, 80.7%, and 56.3%, with a median survival time of 64.0 months in the low-risk group; 98.1%, 56.2%, and 33.9%, with a median survival time of 44.0 months in the moderate-risk group; and 78.6%, 0.0%, and 0.0%, with a median survival time of 23 months in the high-risk group. In Fig. 1B, the difference in the recurrence-free survival rates remained statistically significant between the three groups (*P* < 0.001), with 95.5%, 68.8%, and 40.1% of patients in the low-risk group surviving recurrence-free at 1, 3, and 5 years, with a median time to recurrence of 54.0 months. In the moderate-risk group, the 1-, 3-, and 5-year recurrence-free survival rates were 93.2%, 48.7%, and 24.0%, with a median time to recurrence of 35.0 months. In the high-risk group, the 1-, 3-, and 5-year recurrence-free survival rates were 66.7%, 0.0%, and 0.0%, with a median time to recurrence of 16.0 months.

### The OS and RFS survival of the different risk groups in the validation cohort

**Fig. 2:**
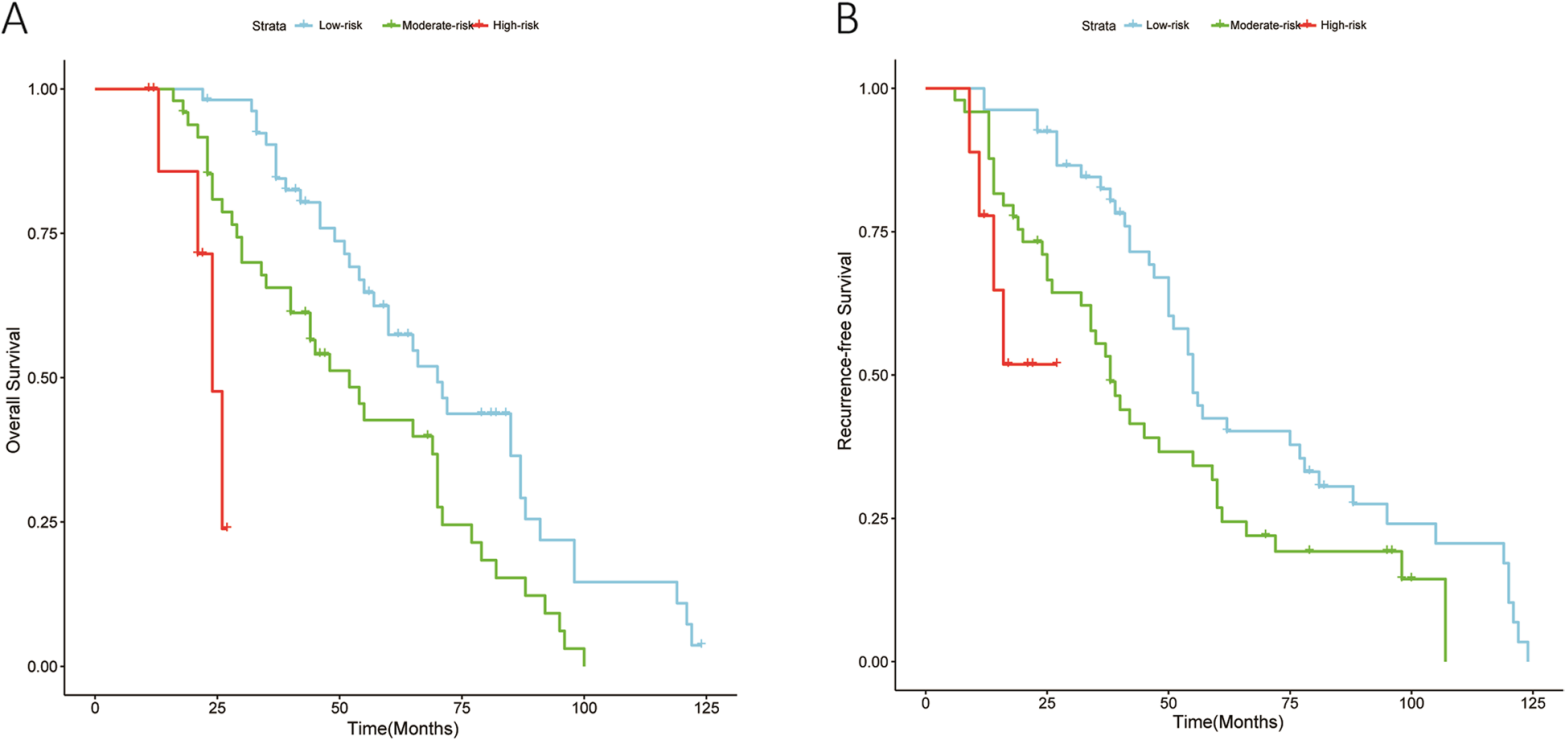
Overall survival (OS) and recurrence-free survival (RFS) of gastric cancer patients in the validation cohort based on MPK scores after surgery. **A** Overall survival. **B** Recurrence-free survival. In the low-risk group, the 1-, 3-, and 5-year overall survival rates were 98.1%, 84.5%, and 57.4%, with a median survival time of 70.0 months. In the moderate-risk group, the corresponding rates were 80.9%, 65.6%, and 0.0%, with a median survival time of 52.0 months. In the high-risk group, the 1-, 3-, and 5-year overall survival rates were 85.7%, 0.0%, and 0.0%, with a median survival time of 24.0 months. The overall survival rates and recurrence-free rates remained statistically significantly different among the three risk groups (*P* < 0.001)

Patients in the validation group were from another gastrointestinal surgery center and were also divided into low, moderate, and high groups according to the MPK score, with 53 patients (47.7%) in the low-risk group, 49 (44.1%) in the moderate-risk group, and 9 (8.1%) in the high-risk group. In Fig. 2A, the OS rates remained statistically significantly different among the three risk groups (*P* < 0.001). The 1-, 3-, and 5-year overall survival rates were 98.1%, 84.5%, and 57.4%, with a median survival time of 70.0 months in the low-risk group; 80.9%, 65.6%, and 0.0%, with a median survival time of 52.0 months in the moderate-risk group; and 85.7%, 0.0%, and 0.0%, with a median survival time of 24.0 months in the high-risk group. Similarly, in Fig. 2B, there was a statistically significant difference in the RFS rates among the three risk groups (*P* < 0.001), consistent with the training group.

### ROC curves for survival of MPK scores at different time periods

**Fig. 3:**
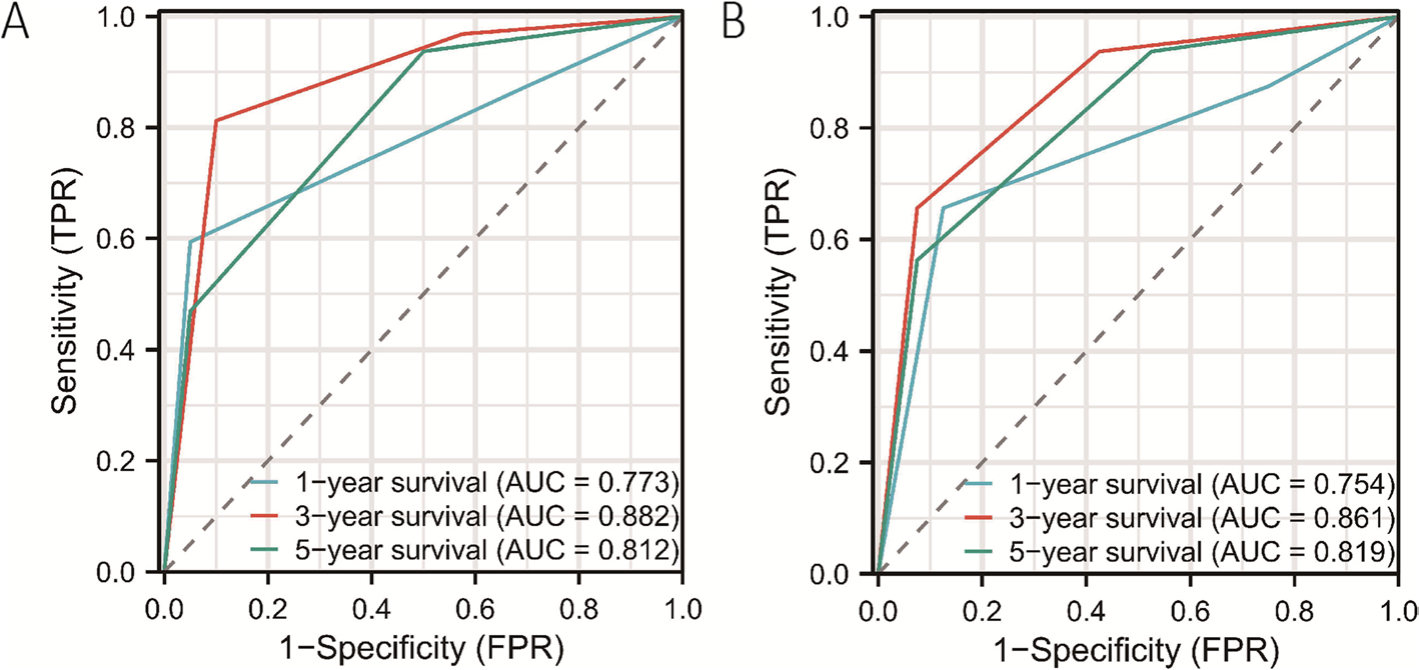
ROC curves for MPK scores. **A** The ROC curves for MPK scores of 1, 3, and 5 years in the training set with the area under the curve of 0.773, 0.882, and 0.812, respectively. **B** The ROC curves for MPK scores of 1, 3, and 5 years in the validation set with the area under the curve of 0.754, 0.861, and 0.819, respectively

In Fig. 3A, the ROC curve areas under the curve (AUC) for 1-, 3-, and 5-year overall survival rates based on the MPK scores in the training cohort were 0.773, 0.882, and 0.812, respectively. In Fig. 3B, in the validation cohort, the AUC values for 1-, 3-, and 5-year overall survival rates based on the MPK scores were 0.773, 0.882, and 0.812, respectively (Fig. 3).

## Discussion

Gastrectomy is undoubtedly one of the best treatments for resectable gastric cancer patients, but there are still difficulties predicting the postoperative outcome of resected gastric cancer patients [14, 15]. Due to the heterogeneity of gastric cancer patients in the current TNM staging system and other stages, there is a large difference in prognosis even if the patients are at the same stage [3]. The current focus should be on developing a more effective scoring system to screen out high-risk patients after surgery and finely manage this group of patients.

We established an immunohistochemical score (MPK score) using the postoperative immunohistochemistry expression data from 245 patients in the training group and validated the model using the validation group. Our multifactorial regression results indicate that MSI status, P53 expression, and Ki-67 expression are important prognostic factors, and these three variables make up the MPK score. For MSI status, which is receiving more attention in recent years [16–18], Filippo et al. [19] conducted a meta-analysis of four multicenter RCTs and showed that MSI is a robust prognostic marker that clinical trials can use to stratify patients and guide chemotherapy. Dudley et al. [4] concluded that MSI not only predicts patient prognosis but also serves as a biomarker to guide the use of immune checkpoint inhibitor (PD-1), and Shen et al. [20] similarly concluded that MSI status plays a vital role in all gastric cancer patients. The results of these investigators are consistent with ours, and MSI has an irreplaceable role in predicting the prognosis and guiding the refinement of therapy.

The P53 protein promoted cell cycle arrest and programmed cell death, and during tumorigenesis, mutations in the P53 gene lead to uncontrolled growth, making it genetically unstable. In various tumor types, P53 mutations are associated with poor prognosis [21, 22]. This is also consistent with our findings that overall survival and recurrence-free survival are poor when patients had positive P53 expression. The accumulation of chronic inflammation can lead to cellular carcinogenesis and tumor progression, in which P53 also plays an important role, and mutations in p53 also affect the expression of other genes. Lee et al. [23] concluded that maspin expression is negatively correlated with mutant P53 expression, P53 regulates maspin expression, and maspin affects the prognosis of patients. In conclusion, P53 gene mutations affect the prognosis of tumor patients in different ways.

Ki-67 is a protein marker related to cell proliferation, and its expression correlates with the depth of invasion and differentiation of various solid tumors, especially with gastrointestinal tumors. Studies on the relationship between ki-67 and prognosis are increasing year by year [24, 25]. Our study indicated that patients have a poorer prognosis when ki-67 expression was ≥ 50%, and many studies agree with us, although they use different cutoff values. A meta-analysis by Luo et al. [12] included more than 5000 patients and showed that high ki-67 expression could be a predictive marker for poor prognosis in gastric cancer patients, and the selection of different cutoff values needs to be considered comprehensively. As with P53, ki-67 also has a vital role in guiding chemotherapy. Chen et al. [26] suggested that high ki-67 is a predictor of pathologic complete response (pCR) in breast cancer patients undergoing neoadjuvant therapy; Yoshikawa et al. [27] suggested that ki-67 could be an effective biomarker for preoperative radiotherapy in rectal cancer.

The MPK score, established by combining the three immunohistochemical indices—namely, MSI status, P53, and Ki-67, showed robust differentiation in terms of prognosis and also performed well in the external validation group. We used this scoring system to screen for high-risk patients, which accounted for about 10% of the training and validation groups. The prognosis of this group was inferior, with all patients in the high-risk group dying within 3 years after surgery and experiencing tumor recurrence within 2 years after surgery. Currently, the treatment strategy for gastric cancer patients before and after surgery is often a “one-size-fits-all” approach [28]. Gastric cancer is limited by the great variation of tumor cells within the tumor, so even if they are in the same TNM stage, the prognosis of patients still varies greatly. In addition, the postoperative treatment can change with the recurrence and metastasis of some tumors. Therefore, we believe a more effective and cost-effective strategy with individualized risk-based detection is needed. Patients found to have a high MPK score (high MSI, P53( +), and Ki-67 ≥ 50% upon postoperative immunohistochemistry) should be put on an intensive surveillance program and subsequently treated early. All three indicators have the function of guiding the decision on whether to do adjuvant therapy or not for patients and combining the three indicators may further enhance their ability to guide adjuvant therapy. Meanwhile, our institution is conducting a study to investigate whether the MPK score can be used as a criterion for postoperative adjuvant therapy.

Several important factors influence the prognosis of gastric cancer. Hou et al. [29] found high expression of CD44 in gastric cancer across multiple databases, while the low expression of CD44 was associated with prolonged OS, progression-free survival (PFS), and post-progression survival (PPS). Chen et al. [30] conducted a multifactor Cox analysis and reported an HR of 1.782 for high CD44 expression, indicating its impact on the OS of gastric cancer patients. Human epidermal growth factor receptor 2 (HER2) is reported to be overexpressed in 10–30% of gastric cancer patients [31]. However, its influence on prognosis remains controversial. Yang et al. [32] suggested that low expression of HER2 might lead to distinct biological characteristics but was not an independent prognostic factor for early-stage gastric cancer’s disease-free survival (DFS) or OS. Jiang et al. [33] demonstrated that the co-expression of Sp1 and HER-2 was associated with poor prognosis in gastric cancer patients. Previous studies have established nomograms based on circulating tumor cells (CTCs) [34], which can predict postoperative OS and recurrence-free survival (RFS) in gastric cancer. CTCs, as a liquid biopsy approach, are gaining attention among researchers. In the future, combining the CTCs with the MPK score could be a promising approach to predict patient prognosis. In our study, all three variables comprising the MPK score had HRs > 2.0, and there was a clear distinction observed in the Kaplan–Meier curves.

To our knowledge, our study is the first to combine several of the most important markers to form a composite immunohistochemistry score. Several previous studies have also established predictive scores or models for postoperative gastric cancer. Kim et al. [35] constructed a scoring system to predict the prognosis of advanced gastric cancer using five variables, including serum neutrophil–lymphocyte ratio, alkaline phosphatase level, and albumin level, which had an AUC of 0.661 for 1-year OS, which was not high and the novelty of these variables was insufficient; Luo et al. [36] used an external database to screen nine relevant glycolytic genes for the construction of predictive scores. However, the authors did not use other data for validation, and the utility was not robust. Postoperative immunohistochemical analysis of tumor tissue and the combination of these powerful immunohistochemical markers can more strongly predict the patients’ OS and RFS. The MPK score we constructed could distinguish all postoperative gastric cancer patients very clearly and achieved very good results in the validation group.

This study still has some limitations; first, this study is retrospective and inherently has some bias; therefore, we will use a prospective design in a future study on MPK scores. Second, we used only one set of external data for validation and may need more external databases for validation in the future.

In conclusion, we established an MPK score using three immunohistochemical metrics validated by an external dataset. The MPK score allowed for the differentiation of postoperative gastric cancer patients, screening out patients with poor prognoses and placing these patients under more refined management.

## Supplementary Information

**Additional file 1:** **Table S1.** Univariate and multivariate analysis of overall survival in gastric cancer (GC) patients who underwent gastrectomy in the training cohort after constructing the score.

**Additional file 2: Table S2.** Baseline characteristics of gastric cancer patients undergoing gastrectomy in the training group in risk groups with different MPK scores (*n* = 245).

## Data Availability

The datasets used and analyzed during the current study are available from the corresponding author on reasonable request.

## Abbreviations

GC: Gastric cancer
ASA: American Society of Anesthesiologists
ECOG: Eastern Cooperative Oncology Group
PS: Performance status
MSI: Microsatellite instability
MSS: Microsatellite stable
CT: Computed tomography
MRI: Magnetic resonance imaging
HR: Hazard ratio
OS: Overall survival
RFS: Recurrence-free survival
