# Supplementary material for: A novel immunohistochemical score predicts the postoperative prognosis of gastric cancer patients
Source: World J Surg Oncol. 2023 Jul 26;21:220. doi: 10.1186/s12957-023-03113-7 (PMC10369746; doi:10.1186/s12957-023-03113-7)
Supplement: Supplementary file 1 — Additional file 1: Table S1. Univariate and multivariate analysis of overall survival in gastric cancer (GC) patients who underwent gastrectomy in the training cohort after constructing the score. [file 12957_2023_3113_MOESM1_ESM.docx]

Supplementary Table 1. Univariate and multivariate analysis of overall survival in Gastric cancer (GC) patients who underwent gastrectomy in the training cohort after constructing the score.

|  | **Univariate analysis** | | | **Multivariate analysis** | | |
| --- | --- | --- | --- | --- | --- | --- |
|  | P | HR | 95% confidence interval | P | HR | 95% confidence interval |
| **Gender**  male/female | 0.921 | 1.020 | 0.692-1.504 |  |  |  |
| **Age**  >60 y/≤60 y | 0.181 | 0.817 | 0.608-1.099 |  |  |  |
| **ASA**  II/I | 0.714 | 0.920 | 0.587-1.440 |  |  |  |
| **ECOG PS**  1/0 | 0.984 | 1.004 | 0.706-1.427 |  |  |  |
| **TNM stage** | **<0.001** | 1.711 | 1.490-1.965 | <0.001 | 1.584 | 1.365-1.837 |
| **Tumor size** | **0.002** | 1.594 | 1.182-2.149 | 0.119 | 1.283 | 0.938-1.755 |
| >5.0cm/≤5.0cm |  |  |  |  |  |  |
| **Vascular invasion** | 0.427 | 1.131 | 0.835-1.531 |  |  |  |
| Yes/No |  |  |  |  |  |  |
| **P53** | **<0.001** | 2.298 | 1.682-3.140 | 0.005 | 1.862 | 1.205-2.880 |
| +/- |  |  |  |  |  |  |
| **Ki-67**  ≥/< | **<0.001** | 2.614 | 1.923-3.555 | 0.027 | 1.606 | 1.055-2.444 |
| **MSI status**  MSI-high/MSS or MSI-low | **<0.001** | 2.710 | 1.880-3.907 | <0.001 | 2.771 | 1.864-4.121 |
| **MPK Score** | **<0.001** | 2.732 | 2.082-3.586 | <0.001 | 2.899 | 2.121-3.846 |

ASA: American Society of Anesthesiologists; ECOG: Eastern Cooperative Oncology Group; PS: performance status; MSI: microsatellite instability; MSS: microsatellite stable; HR: hazard ratio.

TNM stages are according to AJCC 8^th^ edition
