# Supplementary material for: A novel immunohistochemical score predicts the postoperative prognosis of gastric cancer patients
Source: World J Surg Oncol. 2023 Jul 26;21:220. doi: 10.1186/s12957-023-03113-7 (PMC10369746; doi:10.1186/s12957-023-03113-7)
Supplement: Supplementary file 2 — Additional file 2: Table S2. Baseline characteristics of gastric cancer patients undergoing gastrectomy in the training group in risk groups with different MPK scores (n = 245). [file 12957_2023_3113_MOESM2_ESM.docx]

# Supplementary Table 2. Baseline characteristics of gastric cancer patients undergoing gastrectomy in the training group in risk groups with different MPK scores (n = 245)

|  |  | **Low-risk(n=111)** | **Moderate-risk(n=104)** | **High-risk(n=30)** | **P-value** |
| --- | --- | --- | --- | --- | --- |
| Gender(%) |  |  |  |  | <0.001 |
|  | Male | 94(84.7) | 93(89.4) | 17(56.7) |  |
|  | Female | 17(15.3) | 11(10.6) | 13(43.3) |  |
| Age(%) |  |  |  |  | 0.862 |
|  | <60 y | 60(54.1) | 60(57.7) | 17(56.7) |  |
|  | ≥60 y | 51(45.9) | 44(42.3) | 13(43.3) |  |
| ASA(%) |  |  |  |  | 0.953 |
|  | I | 98(88.3) | 92(89.3) | 27(90.0) |  |
|  | II | 13(11.7) | 11(10.7) | 3(10.0) |  |
| ECOG PS(%) |  |  |  |  | 0.684 |
|  | 0 | 85(76.6) | 83(79.8) | 25(83.3) |  |
|  | 1 | 26(23.4) | 21(20.2) | 5(16.7) |  |
| TNM stage(%) |  |  |  |  | <0.001 |
|  | I stage | 41(36.9) | 24(23.0) | 0(0.0) |  |
|  | II stage | 31(27.9) | 27(26.0) | 5(16.7) |  |
|  | III stage | 26(23.4) | 26(25.0) | 9(30.0) |  |
|  | IV stage | 13(11.7) | 27(26.0) | 16(53.3) |  |
| Tumor size(%) |  |  |  |  | 0.210 |
|  | <5 cm | 53(47.7) | 48(46.2) | 9(30.0) |  |
|  | ≥5 cm | 58(52.3) | 56(53.8) | 21(70.0) |  |
| Differentiation(%) |  |  |  |  | <0.001 |
|  | High or moderate | 52(46.8) | 29(27.9) | 5(16.7) |  |
|  | Poor or no | 59(53.2) | 75(72.1) | 25(83.3) |  |
| Vascular invasion(%) |  |  |  |  | 0.576 |
|  | No | 41(36.9) | 32(30.8) | 9(30.0) |  |
|  | Yes | 70(63.1) | 72(69.2) | 21(70.0) |  |
| P53(%) |  |  |  |  | <0.001 |
|  | No | 111(100.0) | 27(26.0) | 0(0.0) |  |
|  | Yes | 0(0.0) | 77(74.0) | 30(100.0) |  |
| Ki-67(%) |  |  |  |  | <0.001 |
|  | <50% | 111(100.0) | 18(17.3) | 0(0.0) |  |
|  | ≥50% | 0(0.0) | 86(82.7) | 30(100.0) |  |
| MSI status(%) |  |  |  |  | <0.001 |
|  | MSS/MSI-low | 110(99.1) | 85(81.7) | 0(0.0) |  |
|  | MSI-high | 1(0.9) | 19(18.3) | 30(100.0) |  |
| Recurrence number | N | 70(63.1) | 86(82.7) | 30(100.0) | <0.001 |
| Recurrence Model |  |  |  |  | 0.030 |
|  | Local recurrence | 12(10.8) | 25(24.0) | 7(23.3) |  |
|  | Lymph node metastasis | 17(15.3) | 11(10.6) | 5(16.7) |  |
|  | Intra-abdominal metastasis | 33(29.7) | 29(27.9) | 8(26.7) |  |
|  | Other organ metastases | 8(7.2) | 21(20.2) | 10(33.3) |  |

ASA: American Society of Anesthesiologists; ECOG: Eastern Cooperative Oncology Group; PS: performance status; MSI: microsatellite instability; MSS: microsatellite stable.

TNM stages are according to AJCC 8^th^ edition
